# Production and characterization of surfactin-like biosurfactant produced by novel strain *Bacillus nealsonii* S2MT and it's potential for oil contaminated soil remediation

**DOI:** 10.1186/s12934-020-01402-4

**Published:** 2020-07-20

**Authors:** Irfan Ali Phulpoto, Zhisheng Yu, Bowen Hu, Yanfen Wang, Fabrice Ndayisenga, Jinmei Li, Hongxia Liang, Muneer Ahmed Qazi

**Affiliations:** 1 College of Resources and Environment, University of Chinese Academy of Sciences, 19 A Yuquan Road, Beijing, 100049 People’s Republic of China; 2 Yanshan Earth Critical Zone and Surface Fluxes Research Station, Chinese Academy of Sciences, No. 380 Huaibei Town, Huairou District, Beijing, 101408 People’s Republic of China; 3 Institute of Microbiology, Faculty of Natural Science, Shah Abdul Latif University, Khairpur Mirκs-66020, Sindh, Pakistan

**Keywords:** *Bacillus nealsonii*, Biosurfactant, Surfactin, Response surface methodology, Bioremediation

## Abstract

**Background:**

Biosurfactants, being highly biodegradable, ecofriendly and multifunctional compounds have wide applications in various industrial sectors including environmental bioremediation. Surfactin, a member of lipopeptide family, which is considered as one of the most powerful biosurfactants due to its excellent emulsifying activities as well as environmental and therapeutic applications. Therefore, the aim of this study was to investigate the newly isolated bacterial strain S2MT for production of surfactin-like biosurfactants and their potential applications for oil-contaminated soil remediation.

**Results:**

In this study, the strain S2MT was isolated from lake sediment and was identified as *Bacillus nealsonii* based on transmitted electron microscopy (TEM) and 16S rRNA ribo-typing. The strain S2MT produced biosurfactant that reduced the surface tension (34.15 ± 0.6 mN/m) and displayed excellent emulsifying potential for kerosene (55 ± 0.3%). Additionally, the maximum biosurfactant product yield of 1300 mg/L was achieved when the composition of the culture medium was optimized through response surface methodology (RSM). Results showed that 2% glycerol and 0.1% NH_4_NO_3_ were the best carbon/nitrogen substrates for biosurfactant production. The parameters such as temperature (30 °C), pH (8), agitation (100 rpm), NH_4_NO_3_ (0.1%) and NaCl (0.5%) displayed most significant contribution towards surface tension reduction that resulted in enhanced biosurfactant yield. Moreover, the extracted biosurfactants were found to be highly stable at environmental factors such as salinity, pH and temperature variations. The biosurfactants were characterized as cyclic lipopeptides relating to surfactin-like isoforms (C^13^–C^15^) using thin-layer chromatography (TLC), Ultra high performance liquid chromatography and mass spectrometry (UHPLC-MS). The crude biosurfactant product displayed up to 43.6 ± 0.08% and 46.7 ± 0.01% remediation of heavy engine-oil contaminated soil at 10 and 40 mg/L concentrations, respectively.

**Conclusion:**

Present study expands the paradigm of surfactin-like biosurfactants produced by novel isolate *Bacillus nealsonii* S2MT for achieving efficient and environmentally acceptable soil remediation as compared to synthetic surfactants.

## Background

The development of sustainable technology has driven the search for natural and biodegradable compounds to remediate sites contaminated with hydrocarbons. This has led to the discovery of surfactants from natural sources. The compounds have surface-active properties and one produced by microbes is termed as biosurfactant [1]. These biomolecules are produced by various microorganisms like bacteria, fungi, and yeast [2, 3]. The biomolecules consist of hydrophobic as well as hydrophilic moieties [4]. Hydrophobic ‘tail’ is a hydrocarbon chain containing saturated/unsaturated and hydroxylated fatty alcohols or fatty acids and the hydrophilic ‘head’ is a polar group that contains mono, oligo or polysaccharides and peptides [5].

Moreover, biosurfactants are also valued better than synthetic counterparts for their low-toxicity, higher biodegradability, environment-friendly nature, increased surface activity, low critical micelles concentration (CMC), active at very low concentrations and stability at extreme conditions like pH, salinity and high or low temperature [6–8]. Due to such unique functional properties and eco-friendly nature, these biological surfactants are expected to become multi-functional constituents of the twenty-first century. Additionally, they have more applications in industrial sectors such pharmaceutical, textile processing, agricultural, cosmetics, personal care, food industries, and environmental applications like soil remediation, hydrocarbon degradation and oil recovery [4, 9–11]. Biosurfactant can be produced by numerous bacterial species. Among them, *Bacillus* species are well known for lipopeptide types of biosurfactants [12]. Lipopeptides are among the most powerful biosurfactants due to their therapeutic properties such as broad-spectrum antimicrobial activity, antiviral and antitumor activity [12, 13].

In general, surfactin, iturin, lichenysin*,* and fengycin are the most important members of the lipopeptide family [13, 14]. Amongst, surfactin is considered the most effective member because of its capability of surface tension reduction of water from 72 to 27 mN/m, efficient emulsification activity, and potential environmental applications such as soil remediation [12]. Despite the countless benefits of lipopeptide biosurfactant over synthetic agents, it is still hindered, and not yet employed on a large scale, mainly due to the high cost of raw material, low product yield and extensive purification processes [7, 12, 15]. A number of efforts have been made recently to overcome the above factors by using cheap or cost-effective material, media optimization, and selection of potential microbes for biosurfactant production [7, 12, 15]. Although, these efforts still remained unsuccessful to generate commercially feasible and profitable lipopeptide production, and will be unable to do so unless the yield of the final product is significantly high from naturally producer microbes [16]. Literature suggested that, in an aquatic ecosystem, the lakes provide diverse ecological habitats, [17] and are considered as most important reservoirs for novel microorganisms capable of producing industrial as well as biotechnological molecules for example antibiotics, extracellular compounds, i.e. enzymes and exopolysaccharides, and their use in environmental applications like bioremediation [17, 18]. However, very limited reports have been highlighted related to biosurfactant producing microbes from the sediment of lakes.

It is well understood that oil-contaminated sites are highly polluted with huge quantities of organic pollutants, whereas the petrochemicals are types of decomposed fossils. We assume that the sediments as a graveyard of dead organic matter and was supposed to carry high loads of organic-contaminant degrading microbial diversity. However, the biosurfactant-production is also a key strategy of microbes to degrade organic pollutants. Therefore, the objective of this study was to investigate the novel bacterial isolate for the production of surface active biomolecules from the Yanqi lake and their potential applications for oil-contaminated soil remediation.

In this work, an efficient biosurfactant-producing strain *Bacillus nealsonii* S2MT was isolated from the sediment of Yanqi Lake, Beijing, China (i.e. an under-explored reservoir). Initially, the isolate was characterized by morpho-microscopic and molecular typing, and then validated for biosurfactant production and medium optimization through design of experiments approach like RSM using Design Expert® software. Furthermore, the obtained crude biosurfactant product of *Bacillus nealsonii* S2MT was chemically characterized by TLC and UHPLC/MS techniques. The product stability to different environmental factors such as pH, temperature and salinity as well as the potential of crude biosurfactants for oil contaminated soil remediation was also evaluated.

## Materials and methods

### Chemicals, reagents, and solvents

In the present study, all the chemicals, reagents, and solvents used were of analytical grade. Penzz oil (Pennzoil Products Company, Houston, TX), Kerosene was purchased from the local market in Beijing, China.

### Site description and sample collection

The sampling site was selected Yanqi lake, located in the southeast region of Huairou District, Beijing (Latitude: 40°23′35.63" Longitude: 116°40′0.43"). The samples were collected from the different layers of lake (4 samples from each), i.e. surface water, deep water (6 m) and sediments, using a special sampler (JC-800 deep water sampler) in sterilized bottles. Water quality parameters such as water temperature (T), dissolved oxygen (DO), total dissolved solids (TDS), pH and salinity were measured in situ with a multi-parameter water quality monitoring Sonde (YSI 6600V2, USA) (Additional file 1: Table S1). All samples were labelled, and stored in refrigerator condition under − 21 °C for further analyses.

### Isolation and screening of biosurfactants producing bacteria

Collected sample were diluted using serial dilution method on surface of Luria bertani (LB) media plates for viable count [19]. The plates were incubated at 28 ºC for 48 to 72 h. After incubation, colonies with different morphology were selected and sub-cultured on new LB plates further to obtain pure organisms. Furthermore, all the isolated bacterial cultures (individually) were screened for biosurfactants production under shake flask condition in minimal salt medium (MSM). Each bacterial isolate was inoculated in 50 mL LB broth, and incubated in shaking incubator 140 rpm for 14–18 h at 28 °C for inoculum preparation. And then, 2% (v/v) inoculum (OD at 600 nm ~ 1.00) was inoculated into 250 mL conical flask containing 100 mL sterilized MSM broth according to Qazi et al. [20]. Briefly, the MSM broth comprised of Na_2_HPO_4_ 2.2 g/L, KH_2_PO_4_ 1.4 g/L, MgSO_4_.7H_2_O 0.6 g/L, FeSO_4_.7H_2_O 0.01 g/L, NaCl 0.3 g/L, CaCl_2_ 0.02 g/L, and 0.1% trace elements solution containing; ZnSO_4_.7H_2_O 2.32 g/L, MnSO_4_.4H_2_O 1.78 g/L, H_3_BO_3_ 0.56 g/L, CuSO_4_.5H_2_O 1.0 g/L, NH_4_MoO_4_.2H_2_O 0.39 g/L and KI 0.66 g/L. Glycerol 2% (v/v) and NH_4_NO_3_ 0.1% (w/v) were used as carbon and nitrogen substrates, respectively, for the growth and biosurfactant production. Medium pH was adjusted at 7.02 ± 02 using 1 M HCl and 1 M NaOH before inoculation, and after inoculation it was subjected to incubation under shaking environment (140 rpm at 28 °C for 5 days). The bacterial density (OD at 600 nm ~ 1.00) was determined using UV-Spectrophotometer (Unico Instrument Co., Ltd., Shanghai, China). During the late stationary phase, the culture broths were taken out and centrifuged at 10,000 rpm for 15 min at 4 °C to collect the cell-free supernatants for the determination of biosurfactant activity as mentioned below in the methods Sect. 2.4.

### Determination of biosurfactant production

#### Oil displacement activity (ODA)

The ODA test was performed according to the method of Datta et al. [21] with minor modifications. 100 µL of used engine oil was placed on the surface of the Petri plate containing 40 mL of distilled water (Milli-Q). After that, 10 µL of cell-free broth was dropped on oil-coated thin film, and the zone of oil displaced was measured.

#### Drop collapse technique (DCT)

Pennzoil (Pennzoil Products Company, Houston, TX), 2 µL was placed on the lid of 96 well plate. The plate was left for equilibration for 1–2 h. After that, 5 µL cell-free supernatants were applied to check the potential of biosurfactant. After one minute, the positive result indicated when the drop of supernatants became collapsed [22].

#### Emulsification index (EI_24%_)

This is another method for screening biosurfactant-producing microorganisms. The cell-free supernatants (2 mL) with an equal amount of kerosene were mixed on the vertex mixer for 2 min and left undisturbed at room temperature for 24 h. The results were observed as described [21, 23].

1 $${\text{EI}} = \frac{{\text{height of emulsified layer after 24 hour}}}{{\text{total height of liquid}}}{{\times 100}}$$

#### Tensio-activity measurement

The tensio-activity, i.e. surface tension reducing capacity, of the cell-free culture containing biosurfactants was determined with the digital tensiometer (JYW-200B., China) using Du Nouy ring method [24]. Distilled water was used as a standard in this method. All the evaluations were taken in triplicates.

### Molecular identification and phylogenetic analysis

After screening, the potential biosurfactants producing isolate S2MT was selected for molecular identification using 16S rDNA gene sequencing homology. The pure bacterial culture was sent for commercial amplification and 16S rDNA gene sequencing to the company (Majorbio Sanger Bio-pharmTechnology Co., Ltd, Beijing, China). Obtained nucleotide sequences were subjected to Basic Local Alignment Search Tool (BLAST) analysis against closely associated taxa available in the GenBank sequence database of National Center for Biotechnology Information (NCBI) (www.ncbi.nlm.nih.gov). Phylogenetic analysis was conducted using MEGA X [25]. The obtained 16S rDNA gene sequences of the present study were submitted to NCBI GenBank for accession number. In addition, the identified bacterial isolate was routinely maintained on LB agar before each screening experiment. The pure culture stock was maintained on LB agar slants and kept at 4 °C for routine use. For long term storage, the pure bacterial cultures were maintained in glycerol broth (≈18% v/v) and kept at − 20 °C.

### Medium optimization on various carbon and nitrogen substrates

Biosurfactant-producing strain S2MT was subjected to grow on different substrates as sole carbon sources such as glucose, glycerol (water-soluble) and kerosene (water-insoluble) with the combination of NH_4_NO_3_, urea and yeast extract as nitrogen sources for the medium optimization. The mineral salts medium (MSM) was supplemented with 2 and 0.1% carbon and nitrogen sources, respectively, and pH was maintained at 7.0 followed by sterilization process at 121 °C for 15 min. The medium was then inoculated with 2% (v/v) prepared inoculum (OD at 600 nm ~ 1.00) and incubated in a shaking incubator (140 rpm at 28 °C) for 5 days.

### Experimental design for statistical screening of critical factors affecting biosurfactant production

After screening the best carbon and nitrogen substrates, the most significant process parameters contributing towards biosurfactants production by the test bacterial strain S2MT were determined. The screening experiments were designed using a statistical approach, a regular 2-level factorial model, for analyzing the main effects of multiple variables in an experiment. The experimental design (as shown in Table 1) comprised of six most critical independent variables (factors) viz. temperature, pH, agitation, NH_4_NO_3_ con., NaCl conc., and yeast extract possibly affecting two dependent variables (responses), i.e. surface tension measurement (mN/m) and biosurfactant product yield.

**Table 1:** Design Summary used in the regular 2-level factorial model of experiments for biosurfactant production

Factor	Name	Units	Type	Level	
				Minimum (−)	Maximum (+ 1)
A	Temperature	°C	Numeric	25	30
B	pH	–	Numeric	6	8
C	Agitation	RPM	Numeric	100	180
D	NH_4_NO_3_ conc	%	Numeric	0.1	1
E	Yeast Extract	%	Numeric	0	0.2
F	NaCl conc	%	Numeric	0.1	0.5

### Recovery of the biosurfactants

For extraction of crude biosurfactant, the extracts were obtained by acid precipitation [26]. Cell-free supernatants were obtained from MSM culture broths (as mentioned above) by centrifuging at 10,000 rpm for 15 min at 4 °C. Supernatants were then acidified by adjusting the pH 2.0 with concentrated hydrochloric acid (HCl) followed by overnight refrigeration at 4 °C. Precipitated cell-free supernatants were again centrifuged at 10,000 rpm for 20 min to collect the pelleted precipitates and pH 7.0 was maintained. Crude biosurfactants were then recovered from the pelleted precipitates through methanol extraction followed by rotary evaporation at 40 °C.

### Product stability to various environmental factors

Biosurfactants stability was monitored at different ranges of temperature, salt concentration, and pH [27]. Thermal stability of crude biosurfactant solution (40 mg/L dissolved in distilled water) was determined at varying temperatures such as (4–121 °C), each for 1 h, followed by cooling at room temperature. The stability of biosurfactant to salt concentration was evaluated by adding NaCl at a concentration of 1–9% (w/v) to the biosurfactant solution. Similarly, the effect of pH was determined at different ranges (3–10) by using 1 M HCl and NaOH to adjust the pH. The stability of biosurfactant was measured in terms of final surface tension measurement after performing triplicate tests.

### Critical micelles concentration (CMC) determination

To examine the CMC of obtained crude biosurfactant, different concentrations (0–100 mg/L) of biosurfactants solution were prepared and subsequently the surface-tension was measured [21].

### Characterization of obtained biosurfactant product

#### Product analysis by thin-layer chromatography (TLC)

Extracted crude biosurfactants from cell-free culture were analyzed by TLC using silica gel 60 plates (Merck CO., Inc., Darmstadt, Germany). Chloroform–methanol-water (65:15:2, v/v) solvent system was used. Various color developing reagents, such as ninhydrin 0.2% in ethanol for lipopeptide with red-pinkish spots, 1–5% H_2_SO_4_ followed by heating at 110 °C for 20 min for glycolipids with brown spots and iodine vapors for lipids, were used to visualize the type of biosurfactants [7].

#### Product analysis by liquid chromatography-mass spectrometry (LC- MS)

Biosurfactant product was dissolved in methanol at 1 mg/mL concentration and filtered (0.22 μm). The electrospray ionization (ESI) mass spectra of the product were analyzed using ultra-high performance liquid chromatography (UHPLC, Ultimate 3000, Dionex) system coupled with TSQ Endura™ Triple Quadrupole Mass Spectrometer (Thermo Scientific, USA). The separation was performed using a Hypersil Gold C_18_, 1.9 μm, 100 × 2.1 mm column. The mobile phase comprised of water, 1% formic acid and acetonitrile. The linear gradient system: 0–3.5 min, 60 to 93% acetonitrile; 3–20 min, keeping 93% acetonitrile and 7% water (1% formic acid); injection volume 5µL; flow rate 0.300 ml/min. The UHPLC-ESI–MS was performed on positive modes with full scans ranging from *m/z* 200 to 2000.

### Potential of obtained biosurfactant in heavy engine oil polluted soil remediation

The efficiency of crude biosurfactants was examined for heavy engine-oil polluted soil remediation. Twenty grams of de-moisturized soil was contaminated with 10% oil in a conical flask. The soil-remediating solution consisted of 60 mL distilled water containing 10 and 40 mg/L (w/v) concentration of the crude biosurfactant (test setup), while the other containing of 10 and 40 mg/L of a chemical surfactant sodium dodecyl sulfate (SDS) and distilled water were used as positive and negative controls, respectively. All the flasks were incubated in a shaking environment (130 rpm for 24 h at 28 °C). After that, the samples were centrifuged at 5000 rpm for 15 min and supernatants were extracted with n-hexane followed by solvent evaporation, and residual oil was measured gravimetrically [12].

### Statistical analysis of experiments

All the experiments related to biosurfactants, functional screening i.e. emulsification, surface tension, and biosurfactant production were performed in triplicates. The mean ± standard deviation (SD) were calculated. The statistical software package, Design Expert® (Version 12), State-Ease, Minneapolis, MN, USA, was used to generate experimental design and perform all the statistical tests for screening of various influential factors affecting biosurfactant production.

## Results and discussion

### Isolation, screening and identification of biosurfactant producing isolate S2MT

**Fig. 1:**
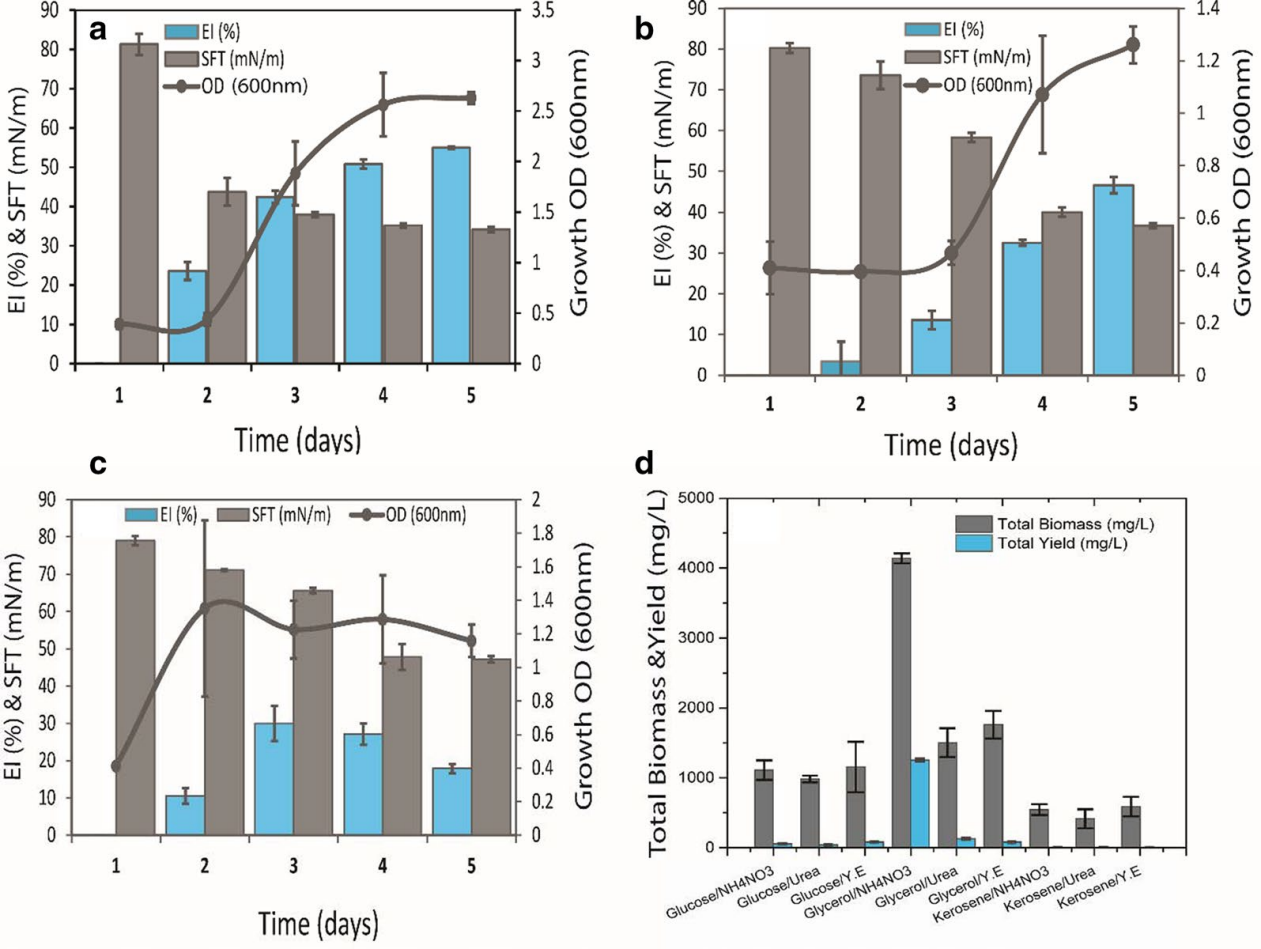
Biosurfactant production profile on fixed glycerol substrate with NH_4_NO_3_ (**a**), urea (**b**), and yeast extract (**c**). Total dry biomass and crude biosurfactant production after five days incubation period at 140 rpm in shaking environment at 28 °C (**d**)

Initially, total of 32 (n = 32) distinct bacterial colonies were isolated from the different layers of lake i.e. surface water, deep water and sediments, and all were screened for their biosurfactants production (Additional file 1: Table S2). Among all, the isolate S2MT was selected as the best biosurfactants producing strain on the basis of different screening methods as mentioned above. The strain S2MT exhibited positive activities in initial biosurfactant screening methods such as efficient activity of biosurfactant product in ODA test displaying clear zone (4.2 ± 0.4 cm) and collapsing of the drop in DCT (Additional file 1: Fig. S1: Table S2). Moreover, the isolate S2MT reduced surface tension (34.15 ± 0.6 mN/m), which was significantly lower than the distilled water, i.e. 81.3 mN/m (Fig. 1a), and expressed excellent emulsification activity (EI = 55 ± 0.3%) for kerosene oil after 24 h. The preliminary identification of isolate S2MT based on morphological and transmitted electron microscopic (TEM) characteristics revealed small, irregular, rough and white colonies that belong to the class *Firmicutes* and genus *Bacillus* (Additional file 1: Fig. S1)*.* After experimental screening and preliminary identification the isolate S2MT was further selected for molecular identification, which was identified as *Bacillus nealsonii*, showing 99.93% similarity using 16S rRNA ribotyping, as illustrated in the phylogenetic tree (Additional file 1: Fig. S2). Nucleotide sequence of 16S rRNA of *B. nealsonii* S2MT strain was submitted in the NCBI GenBank database with accession number (MN128029).

Zhu et al. [28] reported glycerol-based cell-free supernatants showed lowest surface tension value of 27.8mN/m, and highest EI of 38.3% by *B. subtilis* N3-4P, which was comparatively good in surface tension reduction but low emulsifier than current study. According to Cooper and Goldenberg [23], if any bacterial isolate reduces the surface tension (40 mN/m or less), it could be a promising biosurfactant producer. The above results indicate that the strain *Bacillus nealsonii* S2MT is a potential biosurfactant producing strain. Numerous *Bacillus sp*. and related genera have been reported from different types of environments, such as extreme, hydrocarbon-contaminated, terrestrial or marine environment [7], for biosurfactants production by *B. licheniformis*, *Aeribacillus sp*., *Bacillus subtilis* etc. [13, 29]. So far, there are no any previous reports on biosurfactant production by any of the *Bacillus nealsonii* strains.

### Medium optimization on different carbon/nitrogen substrates and product recovery

A number of different C/N sources (as mentioned above in methods) were provided to improve the biosurfactant product yield. It was observed that the C/N combination of glycerol/NH_4_NO_3_, significantly contributed to produce maximum biosurfactant yield, while achieving the lowest SFT (34.15 ± 0.6 mN/m) and maximum EI (55 ± 0.3%) (Fig. 1a). Whereas, the total dry biomass and crude biosurfactant yield were 4140 ± 70.71 mg/L and 1255 ± 21.21 mg/L, respectively (Fig. 1a). The glycerol/Urea preceded the yeast extract as second most important C/N combination (Fig. 1b, c), which resulted in efficient surface tension reduction (36.7 ± 0.6 mN/m) and 46.6 ± 2% emulsification activity together with total dry biomass 1505 ± 205.0 mg/L and crude biosurfactant yield up to 130 ± 13.43 mg/L (Fig. 1d). However, other combinations of C/N sources such as glucose with yeast extract, urea, and even NH_4_NO_3_ did not alter biosurfactant production to significant level except increase in cellular biomass, while kerosene acted as an inhibitor due to its toxicity (Additional file 1: Fig. S3a–i, except d, f). All this together suggested that the combination of glycerol (2%, v/v) and NH_4_NO_3_ (0.1%, w/v) was the most effective C/N source for biosurfactant production by strain *B. nealsonii* S2MT in comparison to glucose and kerosene substrates.

Previous findings also demonstrated that the NH_4_NO_3_ with various carbon substrates have been found an efficient nitrogen source for biosurfactant production by *Bacillus* species*.* Medeot et al. [30] achieved the maximum biosurfactant concentration (1.7 mg/mL) by *Bacillus amyloliquefaciens* MEP218 upon using the glucose and NH_4_NO_3_. Likewise, Fernandes et al. [31] reported NH_4_NO_3_ combined with sucrose to get the highest concentration of biosurfactant by *Bacillus subtilis* RI4914 (0. 2 g/L). Also, Abdel-Mawgound et al. [32] studied surfactin production by *Bacillus subtilis* BS5 using different carbon and nitrogen sources and concluded the highest biosurfactant production in NaNO_3_ and NH_4_NO_3_. Evidently, nitrogen source play a crucial role in biosurfactant production [15], but it depends upon the carbon/nitrogen substrates combination.

### Statistical screening of critical factors for biosurfactant production

The results of multi-parameter screening experiments showing the effects of 6 factors on 2 responses, i.e. SFT reduction and product yield, by combining them in different proportions by the model are mentioned (Table 2). Minimum SFT values 33.7, 34.2, 34.3, and 34.5 mN/m and product yield of 1300, 1110, 900, and 850 mg/L were observed in Run nos. 10, 7, 13 and 19, respectively. Contrarily, the increased value of SFT was found 64, 49.5, 46.8 and 44.5 in Run nos. 14, 20, 11 and 17, respectively. Moreover, the product recovery was only 10, 15, 60, and 60 mg/L by the same experimental runs. It was thus found that surface tension reduction was directly proportional to the product yield.

**Table 2:** Design layout of a regular 2-level factorial model showing different influential factors and their effect on Surface tension and biosurfactants product yield

Run	Factor1 A: Temp (°C)	Factor2B: pH	Factor3 C: Agitation	Factor4 D: NH4NO3 (%)	Factor5 E: Yeast extract (%)	Factor6 F: NaCl (%)	Response1: SFT (mN/m)	Response2: Yield (mg/L)
1	25	8	180	1	0	0.5	37.7	80
2	27.5	7	140	0.55	0.1	0.3	37.4	400
3	25	6	180	0.1	0.2	0.5	34.9	380
4	27.5	7	140	0.55	0.1	0.3	37.6	390
5	30	6	180	0.1	0	0.5	34.9	380
6	25	8	100	1	0.2	0.1	36.5	60
7	25	8	100	0.1	0.2	0.5	34.2	1110
8	27.5	7	140	0.55	0.1	0.3	36.8	440
9	30	8	180	1	0.2	0.5	37.6	60
10	30	8	100	0.1	0	0.5	33.7	1300
11	25	6	100	1	0	0.5	46.8	60
12	30	6	100	1	0.2	0.5	37.6	30
13	30	8	180	0.1	0.2	0.1	34.3	900
14	25	6	100	0.1	0	0.1	64.6	10
15	27.5	7	140	0.55	0.1	0.3	37.1	380
16	30	6	100	0.1	0.2	0.1	35.4	50
17	30	8	100	1	0	0.1	38.2	60
18	25	6	180	1	0.2	0.1	38.1	61.6
19	25	8	180	0.1	0	0.1	34.5	830
20	30	6	180	1	0	0.1	49.5	15

The analysis of variance and regression of both responses, i.e. R1 = SFT (mN/m) and R2 = yield (mg/L), gave details of the most significant terms of the model (Additional file 1: Table S3). As per ANOVA summary, the model was highly significant (*P* < 0.05) to elucidate the effect of significate model terms that affected SFT and yield. The most significant factors were pH, temperature, yeast extract and NaCl conc., with *P* values (*P* = 0.0028), (*P* = 0.0036), (*P* = 0.0042) and (*P* = 0.0319), respectively. Whereas, the most significant factors with *P* values on yield were NH_4_NO_3_ (*P* = 0.0005), temperature (*P* = 0.0039) and pH (*P* = 0.0044). The normal probability chart and the Pareto chart indicated the rank-wise positive and negative effects of factors on biosurfactant production based on the model responses (Additional file 1: Fig. S4). However, the effects of individual factors of significant model terms on biosurfactant production are depicted (Additional file 1: Fig. S5).

**Fig. 2:**
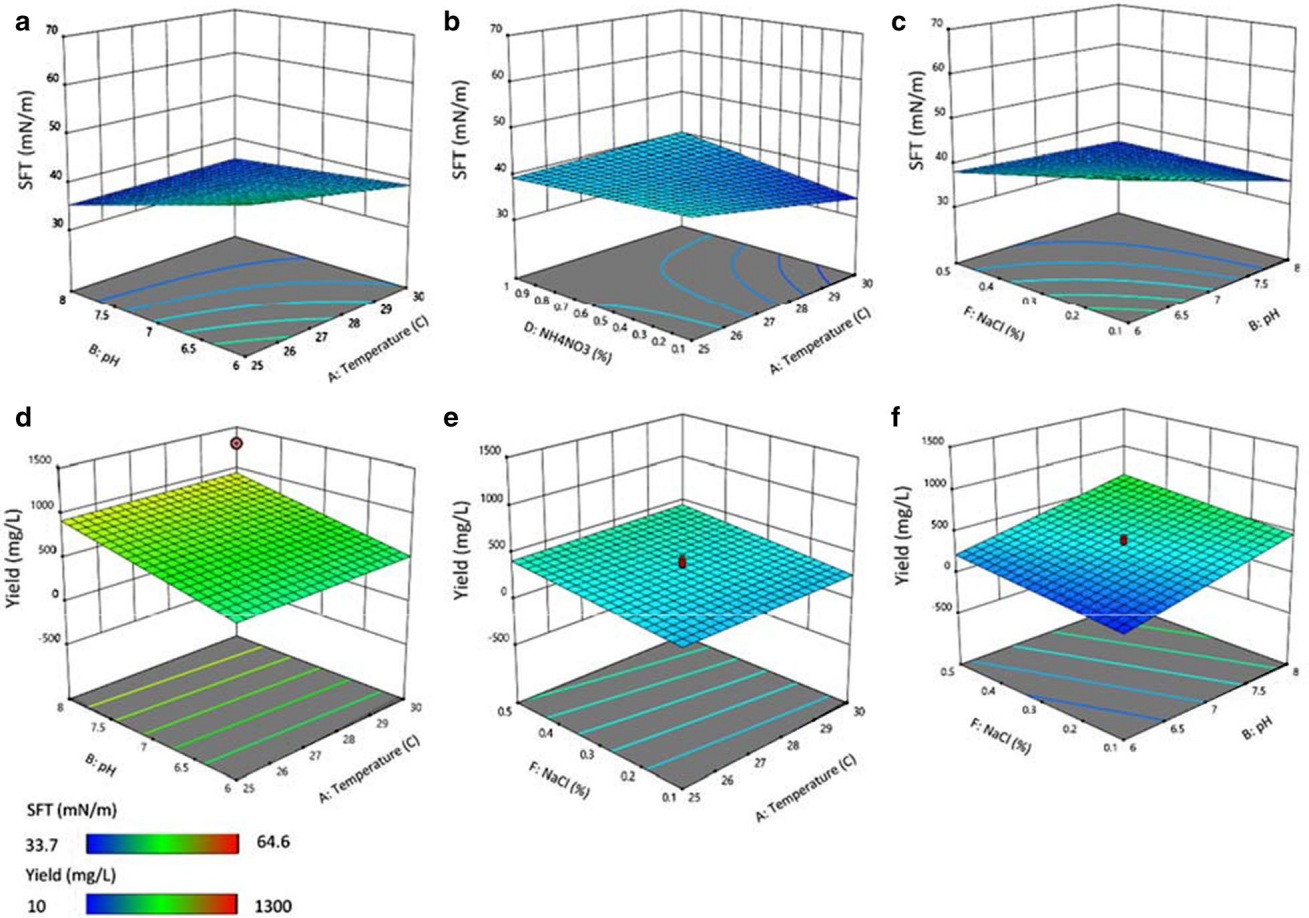
Three dimensional (3D) response surface plots showing the interactive effects of various factors on the surface tension reduction (**a**–**c**) and biosurfactant product yield (mg/L) (**d**, **e**)

Response surface plot and wireframes show the effect of combined factors i.e. pH *vs* temperature, NH_4_NO_3_*vs.* temperature, NaCl *vs.* temperature and NaCl *vs.* pH on SFT reduction and biosurfactants yield (Figs. 2a–f). Figures 2a, d show the decreased SFT value and maximum biosurfactant yield, when the pH and temperature were increased. The increased pH (8) and high temperature (30 °C) may have a significant effect on biosurfactants, whereas a decreased of both factors may have an insignificant effect on biosurfactant production [33]. The NH_4_NO_3_ (0.1%) and high temperature (30 °C), and increased pH (8) and NaCl con. (0.5%) also showed a positive effect on both responses R1 and R2 (Fig. 2b, c, f). Similarly, high NaCl conc. (0.5%) and high temperature (30 °C) had significant effect on biosurfactant production (Fig. 2e), whereas, low NaCl conc. and high temperature may also have a negative effect on biosurfactants [33]. The experimental design (v. 12), based on the regular two-level factorial model suggested the equation for increased biosurfactant production as

2 $$Yield = { 349}.{83 } + { 12}.{71 }\left( {\text{A}} \right) \, + { 213}.{37 }\left( {\text{B}} \right) \, + { 1}.{66 }\left( {\text{C}} \right) \, - { 283}.{34 }\left( {\text{D}} \right) \, - { 5}.{21 }\left( {\text{E}} \right) \, + { 88}.{34 }\left( {\text{F}} \right)$$

where, the A, B, C, D, E, and F designate coded values for model terms temperature, pH, agitation, NH_4_NO_3_, Yeast extract, and NaCl, respectively. After optimization conditions, the biosurfactant production was increased 3.58% more, than initial prodcution by strain S2MT.

### Stability of biosurfactant production

**Fig. 3:**
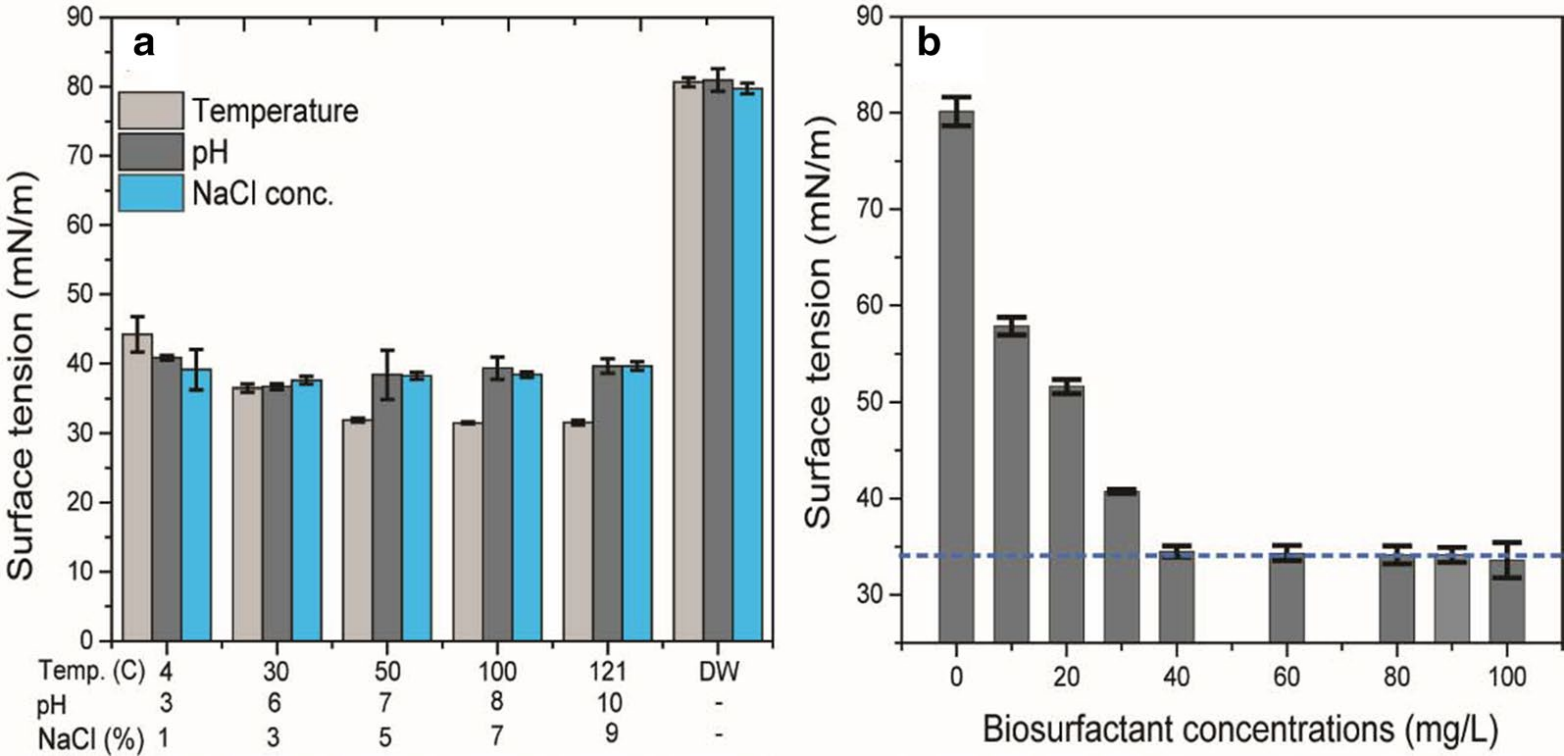
Stability of crude biosurfactant on various environmental factors i.e. temperature ranges 4–121 °C, NaCl conc. 1–9% (w/v), and pH ranges 3–10 (**a**). Critical micelles concentration profile of different concentrations of crude biosurfactant 0–100 mg/L (**b**). (Abbreviation: *DW* distilled water)

The effect of environmental factors on crude biosurfactants is shown in Fig. 3a and Additional file 1: Table S4. As a result of temperature variation, the SFT was found to decrease (31.5 ± 0.1 mN/m) at 100 °C, however, when the temperature decreased as low as 4 °C, the surface tension was found to increase up to 44.2 ± 2.5 mN/m. The biosurfactant product of S2MT strain was found highly stable at high temperature. The surface tension was found to be increased at pH 3 as 40.8 ± 0.4mN/m, whereas, the value was decreased 36.7 ± 0.4 mN/m at 6 pH. The acidic condition was not in the favor of biosurfactant’s stability but it could be due to the precipitation of biosurfactants [12, 34]. In the case of NaCl concentration, slight differences were found in SFT measurement; since the elevated salt concentrations gradually increased the SFT values from 37.6 ± 0.6 mN/m to 39.65 ± 0.0.6 mN/m at 3 and 9% of NaCl concentrations, respectively. The reason for such increase in SFT values might be the ionic salts form ion–dipole interactions with water, which is stronger than the gaseous phase and salt interactions, and causing the solute molecules to avoid the interface [12].

### Critical micelles concentration (CMC) determination

The result of CMC is shown in Fig. 3b. Surface-tension value of various biosurfactant solutions (0–100 mg/L) were recorded. The CMC value of the obtained biosurfactant product was found to be at 40 mg/L, where the SFT was reduced from 81.2 mN/m to 34.5 ± 0.56 mN/m and later there were no significant change in the SFT measurement. The present results of CMC were in agreement with Datta et al. [21], who observed CMC (40 mg/L) by *Bacillus subtilis* MG495086.

### Product characterization

The crude biosurfactant of *Bacillus nealsonii* strain S2MT was analyzed on the TLC plate that indicated lipopeptide in nature. Different spots at Rf values 0.25, 0.35 and 0.75 were observed after spraying with 0.2% ninhydrin reagent as illustrated (Additional file 1: Fig. S6). The lipopeptides might be most related to the surfactin family of the lipopeptides. Similar results were obtained by Ramyabharathi and co-authors on *Bacillus subtilis* Bbv57 producing surfactin and iturin, which were confirmed on TLC with Rf value of 0.3 for surfactin and 0.7 for iturin family while comparing with the standard (Sigma-Aldrich) [35]. Likewise, Yánez-Mendizábal and co-workers reported surfactin and iturin with Rf values 0.3 and 0.7 respectively [36]. Joy et al. [7] also reported similar pattern of TLC with an Rf value of 0.72 and 0.55 for lipopeptide biosurfactants produced by *Bacillus sp.* (SB2).

**Fig. 4:**
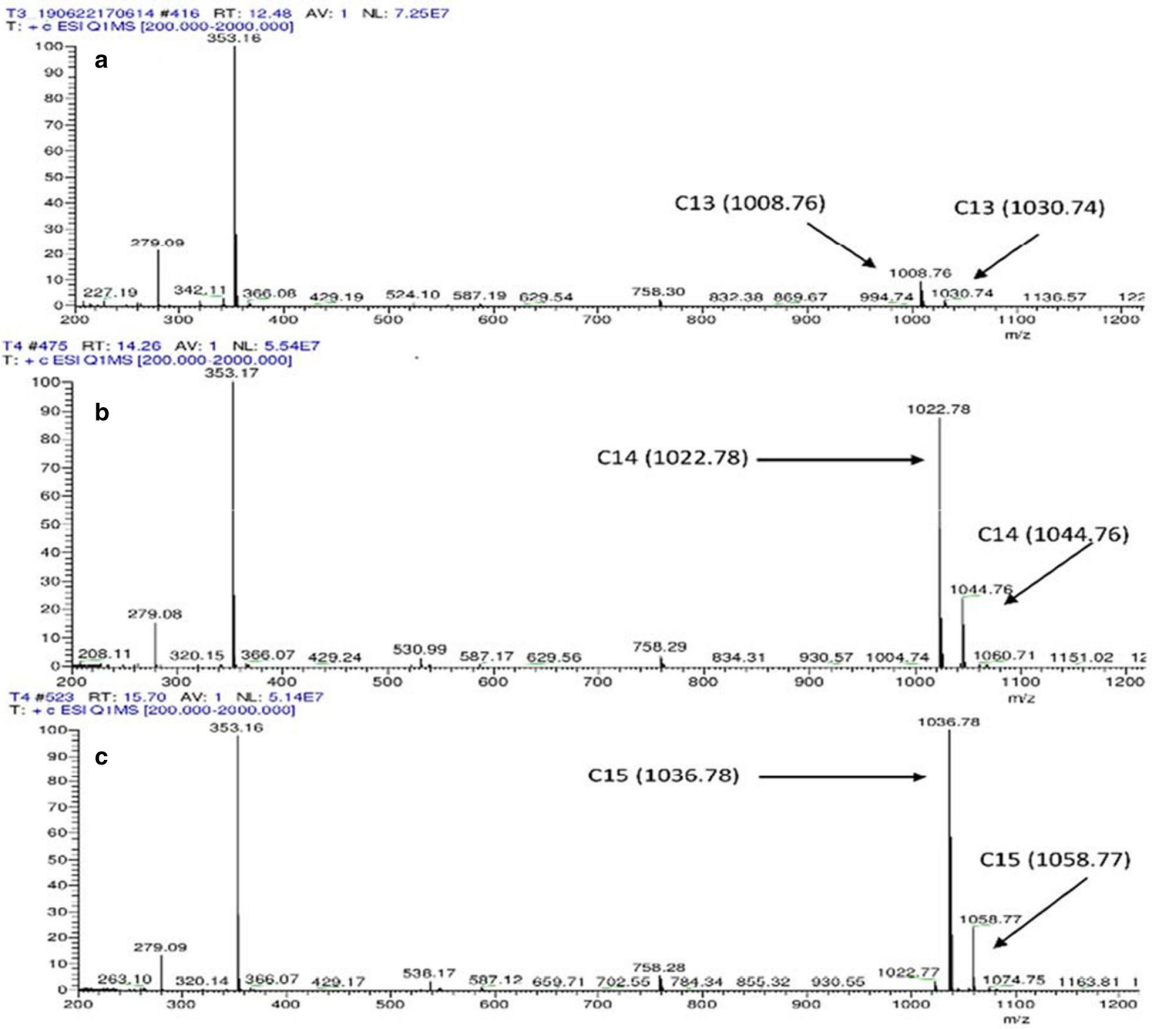
UHPLC-ESI/MS spectra of lipopeptide biosurfactant (C^13^-^15^). 2 isoforms 1008.76, and 1030.74 with retention time (Rt) 12.48 (**a**), 2 isoforms 1022.78 and 1044.76 with (Rt) 14.26 (**b**) and 3visoforms 1022.77, 1036.78, and 1058.77 with (Rt) 15.7 (**c**) detected on positive scan mode

Furthermore, the biosurfactant product was confirmed by UHPLC-ESI/MS. The results of *m/z* peaks are summarized (Additional file 1: Table S5). In total, 6 different surfactin like isoforms (C^13^-C^15^) of lipopeptides, at *m/z* 1008.76 and 1030.74 with retention time (Rt 12.48); 1022.78 and 1044.75 (Rt 14.26); and 1036.79 and 1058.78 with (Rt 15.70) were detected by LC/MS as shown in the chromatogram (Fig. 4). In this respect, a similar pattern was reported by Li et al. [14] for different surfactin like homologs by *B. licheniformis* HSN221, when cultivated in the MSM medium containing glucose, yeast extract, and ammonium nitrate. de Faria et al. [37] reported purified surfactin biosurfactant through the ESI mass spectra of *m*/*z* 1022, 1036, 1044 and 1058, produced by *Bacillus subtilis* LSFM-05 when grown on raw glycerol. Chen and co-authors also reported surfactin homologs by *B. licheniformis* MB01 at *m/z* 994, 1008, 1022, 1036 with isoforms C^12^, C^13^, C^14^, and C^15^, respectively [13]. The above analyses revealed that the biosurfactant produced by *Bacillus nealsonii* S2MT strain is a lipopeptide in nature having the highest resemblance with the surfactin family. In general, surfactin biosurfactants relating to the family of cyclic lipopeptide are mostly produced by *Bacillus* spp.[29].

### Potential of crude biosurfactant in heavy engine oil polluted soil remediation

**Fig. 5:**
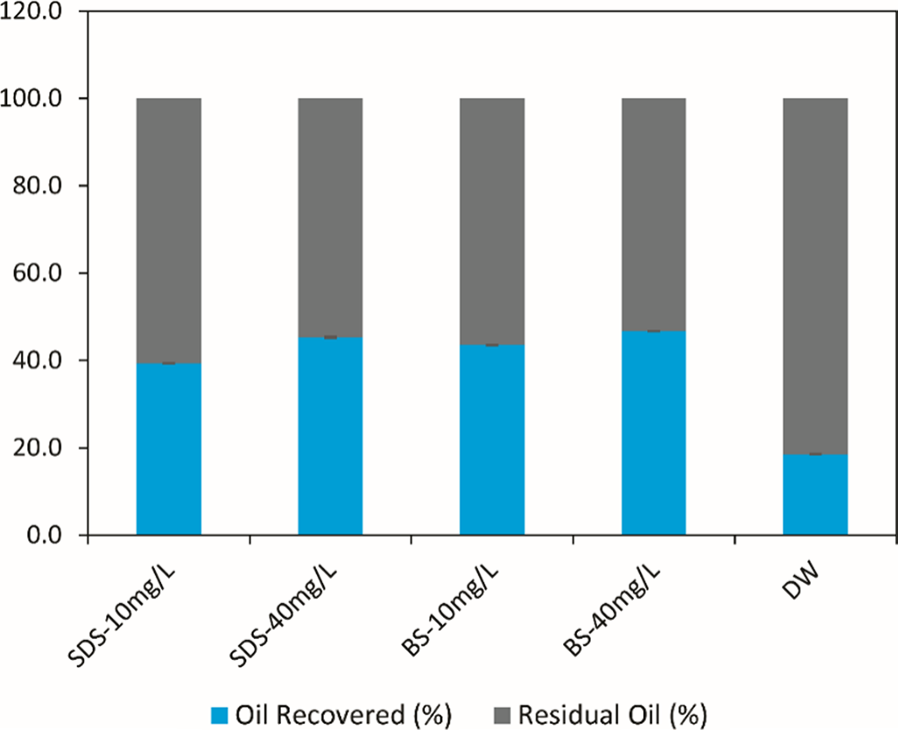
Potential of surfactin-like biosurfactants produced by *Bacillus nealsonii* S2MT for heavy engine oil polluted soil remediation as compare to synthetic surfactants, which shows the percent oil recovery and residual oil from contaminated soil. The abbreviation is the same as used in Fig. 3

Petroleum hydrocarbon contaminants are major social and ecological issues. These contaminants bind to soil particles and are difficult to remove because of their strong sorption and hydrophobicity. Microbial surfactants in oil-polluted soil can emulsify these compounds and enhanced their solubility, decreased surface tension and increased oil displacement from soil particles [12, 26, 38, 39]. Figure 5 illustrates the potential of obtained crude biosurfactant by *B. nealsonii* S2MT in heavy oil-contaminated soil remediation that resulted in 43.6 ± 0.08 and 46.7 ± 0.01% with the concentration 10 and 40 mg/L of crude biosurfactant, respectively. However, with synthetic surfactants, sodium dodecyl sulfate (SDS) obtained 39.4 ± 0.01 and 45.3 ± 0.14% removal of contaminants with the same concentration of 10 and 40 mg/L, respectively. Whereas, only 18.5 ± 0.07% removal of contaminants was achieved with distilled water (Fig. 5).

In this respect, Souza and co-workers obtained 20% remediation of oil-contaminated sand by *Wickerhamomyces anomalus* CCMA 0358 [40], which was lower than the current study. Felix et al. [12] used semi-purified biosurfactant by *B. subtilis* in diesel oil-contaminated soil remediation, and who obtained 78.5% and 81.8% removal with a concentration of 12.5 and 37.5 mg/L. In contrast, the biosurfactants are natural compounds, eco-friendly, non-or-less toxic, biodegradable, more powerful than a synthetic one, and could be used as a crude product in bioremediation applications.

## Conclusion

In the present investigation, a naturally potential biosurfactant producer bacterium *Bacillus nealsonii* S2MT was isolated from the sediment of the Yanqi Lake (first report). Biosurfactants produced by this strain have effective surface tension reducing and emulsifying capabilities in addition to their strong stability over wide range of environmental factors. Interestingly, the strain produced 1255 mg/L crude lipopeptide biosurfactant by utilizing glycerol and NH_4_NO_3_ as carbon and nitrogen sources. The product was improved up to 1300 mg/L when the medium composition was optimized on various factors through RSM approach for designing experiments using statistical software. The CMC values of the obtained product were determined at 40 mg/L. Additionally, the biosurfactant product was confirmed as surfactin, a member of lipopeptide family, through TLC and LC–MS analysis. Furthermore, the biosurfactants produced by strain *Bacillus nealsonii* S2MT has effective heavy engine-oil polluted soil remediation potential and could be used in many other environmental applications.

## Supplementary information

**Additional file 1: Figure S1** Culture morphology of isolated strain S2MT (Bacillus nealsonii) (**a**), TEM image of strain (**b**), and TEM image during biosurfactant production, red arrow indicates the extracellular biomass produced by isolated strain (**c**). Potential of biosurfactant in oil displacement activity (**d**), drop collapse activity (**e**), and emulsification activity (**f**). **Figure S2** Phylogenetic analysis of isolate S2MT with closely related taxa. **Figure S3** Optimization profile of strain B. nealsonii S2MT for various carbon and nitrogen sources for product recovery (**a**–**i**). **Figure S4** Percent contribution of normal probability chart, and Pareto chart indicates the rank wise positive and negative effects factors on biosurfactant production based on model (**a**, **b**). While the interaction between the factors such as temperature vs pH (**c**), temperature vs NaCl (**d**), and pH vs NaCl (**e**). **Figure S5** Significant effects of individual factors i.e. temperature, pH, agitation, NH4NO3, yeast extract and NaCl conc. (**a**–**f**) respectively, on biosurfactant production yield according to model terms. **Figure S6** Thin-layer chromatography analysis of lipopeptide biosurfactant. Various reagents were applied to detect the color development figure (**a**) and the spots were also detect in UV figure (**b**). **Table S1** Physico-chemical and environmental parameters of collected samples. **Table S2** Screening of all 32 cultivable bacterial isolates for biosurfactants production. **Table S3** ANOVA table of the selected regular two-level factorial model designed for SFT (mN/m) reduction and product yield under laboratory settings. **Table S4** Effect of environmental factors (temperature, pH and NaCl concentrations) on (40mg/L) crude biosurfactant production by determining surface tension measurement. **Table S5** Molecular mass study of lipopeptide biosurfactants of Bacillus nealsonii (S2MT) by LC-ESI/MS.
