# Supplementary material for: Production and characterization of surfactin-like biosurfactant produced by novel strain Bacillus nealsonii S2MT and it's potential for oil contaminated soil remediation
Source: Microb Cell Fact. 2020 Jul 20;19:145. doi: 10.1186/s12934-020-01402-4 (PMC7372866; doi:10.1186/s12934-020-01402-4)
Supplement: Supplementary file 1 — Additional file 1: Figure S1 Culture morphology of isolated strain S2MT (Bacillus nealsonii) (a), TEM image of strain (b), and TEM image during biosurfactant production, red arrow indicates the extracellular biomass produced by isolated strain (c). Potential of biosurfactant in oil displacement activity (d), drop collapse activity (e), and emulsification activity (f). Figure S2 Phylogenetic analysis of isolate S2MT with closely related taxa. Figure S3 Optimization profile of strain B. nealsonii S2MT for various carbon and nitrogen sources for product recovery (a–i). Figure S4 Percent contribution of normal probability chart, and Pareto chart indicates the rank wise positive and negative effects factors on biosurfactant production based on model (a, b). While the interaction between the factors such as temperature vs pH (c), temperature vs NaCl (d), and pH vs NaCl (e). Figure S5 Significant effects of individual factors i.e. temperature, pH, agitation, NH4NO3, yeast extract and NaCl conc. (a–f) respectively, on biosurfactant production yield according to model terms. Figure S6 Thin-layer chromatography analysis of lipopeptide biosurfactant. Various reagents were applied to detect the color development figure (a) and the spots were also detect in UV figure (b). Table S1 Physico-chemical and environmental parameters of collected samples. Table S2 Screening of all 32 cultivable bacterial isolates for biosurfactants production. Table S3 ANOVA table of the selected regular two-level factorial model designed for SFT (mN/m) reduction and product yield under laboratory settings. Table S4 Effect of environmental factors (temperature, pH and NaCl concentrations) on (40mg/L) crude biosurfactant production by determining surface tension measurement. Table S5 Molecular mass study of lipopeptide biosurfactants of Bacillus nealsonii (S2MT) by LC-ESI/MS. [file 12934_2020_1402_MOESM1_ESM.docx]

**Production and characterization of surfactin-like biosurfactant produced by novel strain *Bacillus nealsonii* S2MT and it's potential for oil contaminated soil remediation**

Irfan Ali Phulpoto^1^, Zhisheng Yu ^1 *^, Bowen Hu ^1^, Yanfen Wang^2^, Fabrice Ndayisenga ^1^, Jinmei Li^1^, Hongxia Liang^1^, and Muneer Ahmed Qazi^3^

*^1^*College of Resources and Environment, University of Chinese Academy of Sciences, 19 A Yuquan Road, Beijing 100049, P.R. China

^2^ Yanshan Earth Critical Zone and Surface Fluxes Research Station, Chinese Academy of Sciences, No. 380 Huaibei Town, Huairou District, Beijing 101408，P.R. China

*^3^*Institute of Microbiology, Faculty of Natural Science, Shah Abdul Latif University, Khairpur Mir's - 66020, Sindh, Pakistan.

*Corresponding Author(s):

**Prof. Dr. Zhisheng Yu**

College of Resources and Environment, University of Chinese Academy of Science, 19 A Yuquan Road, Beijing 100049, P.R. China

Email: [yuzs@ucas.ac.cn](mailto:yuzs@ucas.ac.cn)

**Supplementary Information**

12 Pages including cover page

6 Figures

5 Tables

**
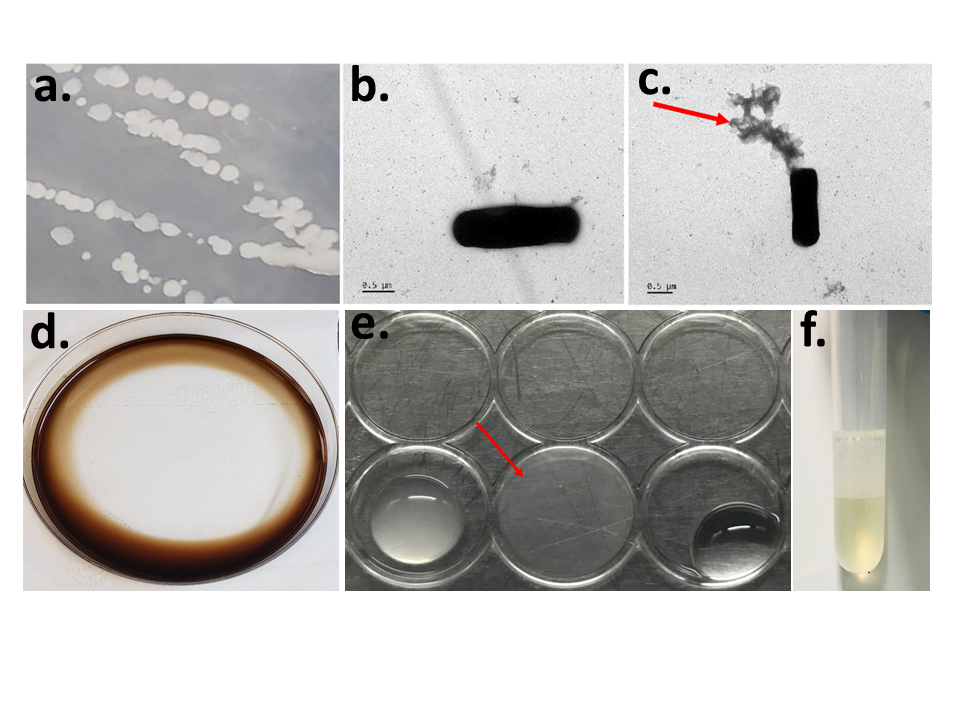
**

**Figure S1.** Culture morphology of isolated strain S2MT (*Bacillus nealsonii*) (a), TEM image of strain (b), and TEM image during biosurfactant production, red arrow indicates the extracellular biomass produced by isolated strain (c). Potential of biosurfactant in oil displacement activity (d), drop collapse activity (e), and emulsification activity (f).

**

**

**Figure S2.** Phylogenetic analysis of isolate S2MT with closely related taxa.


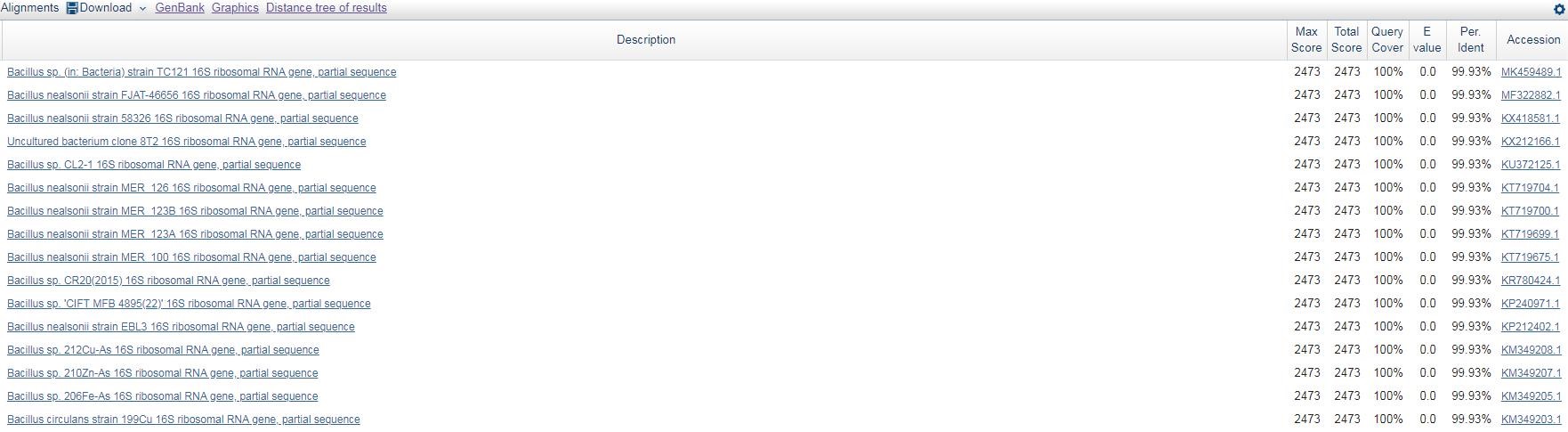


A snapshot of closely related texa with isolate S2MT on BLAST search tool showing query coverage and percent identification.

­­­­­
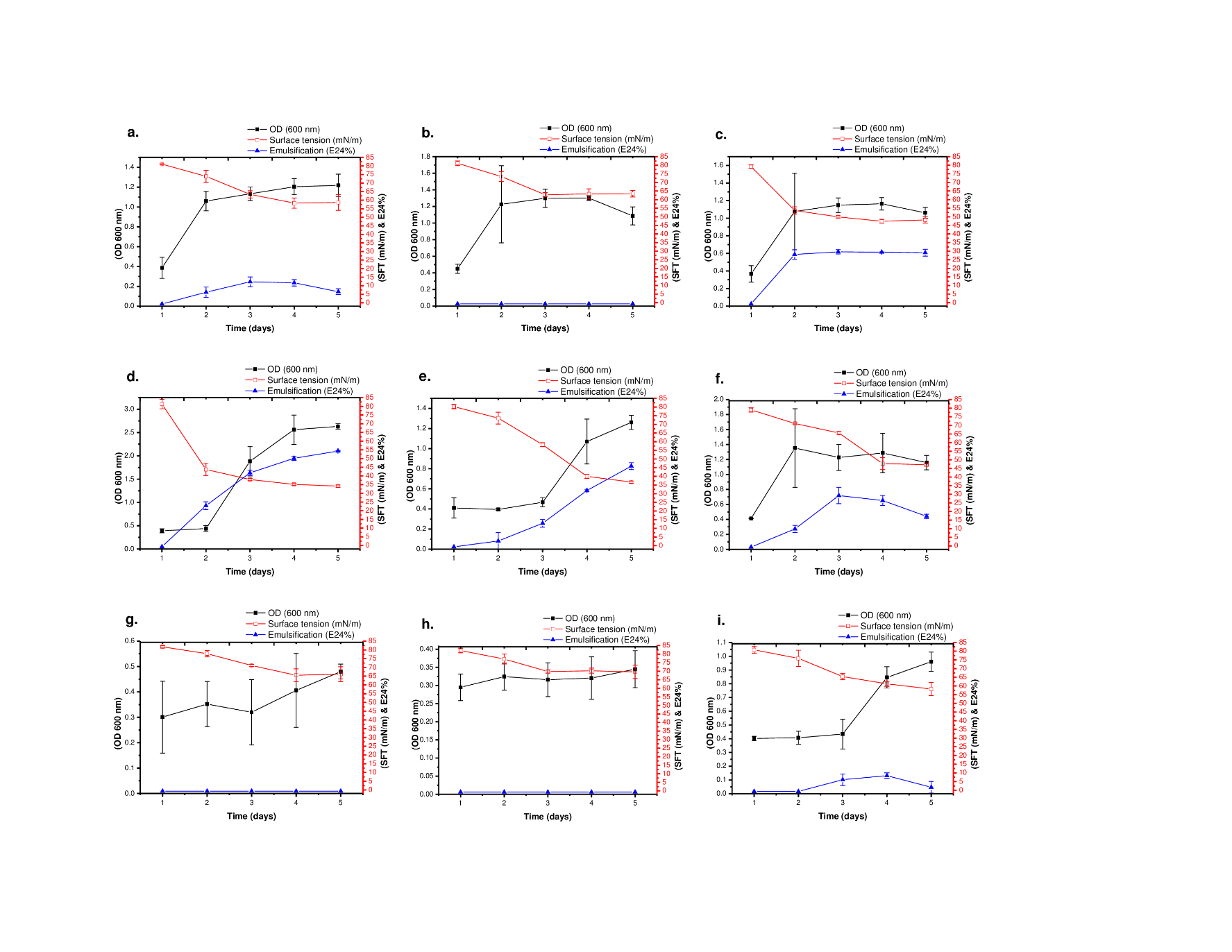


**Figure S3.** Optimization profile of strain *B. nealsonii* S2MT for various carbon and nitrogen sources for product recovery (a-i).

**
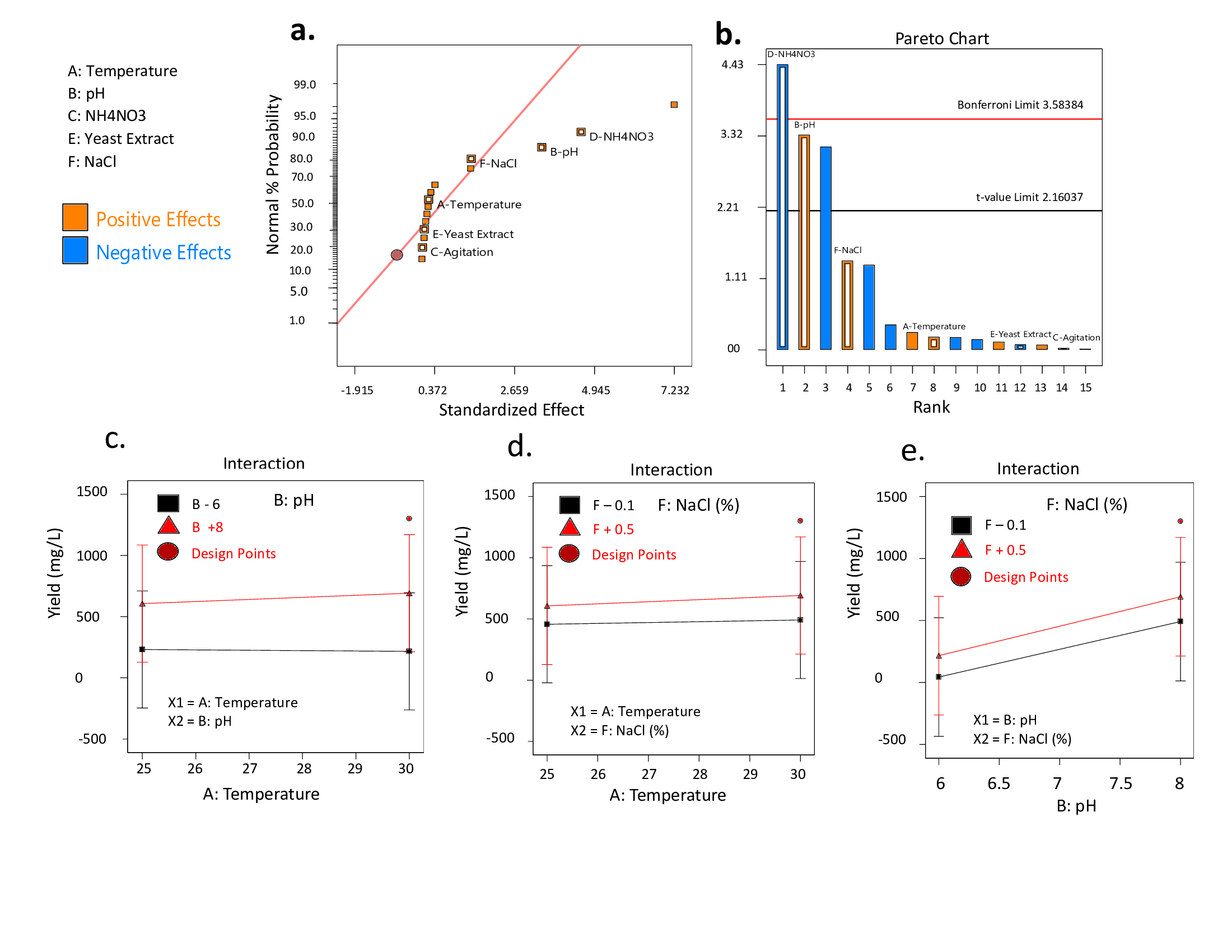
**

**Figure S4.** Percent contribution of normal probability chart, and Pareto chart indicates the rank wise positive and negative effects factors on biosurfactant production based on model (a &b). While the interaction between the factors such as temperature *vs* pH (c), temperature *vs* NaCl (d), and pH *vs* NaCl (e).

**
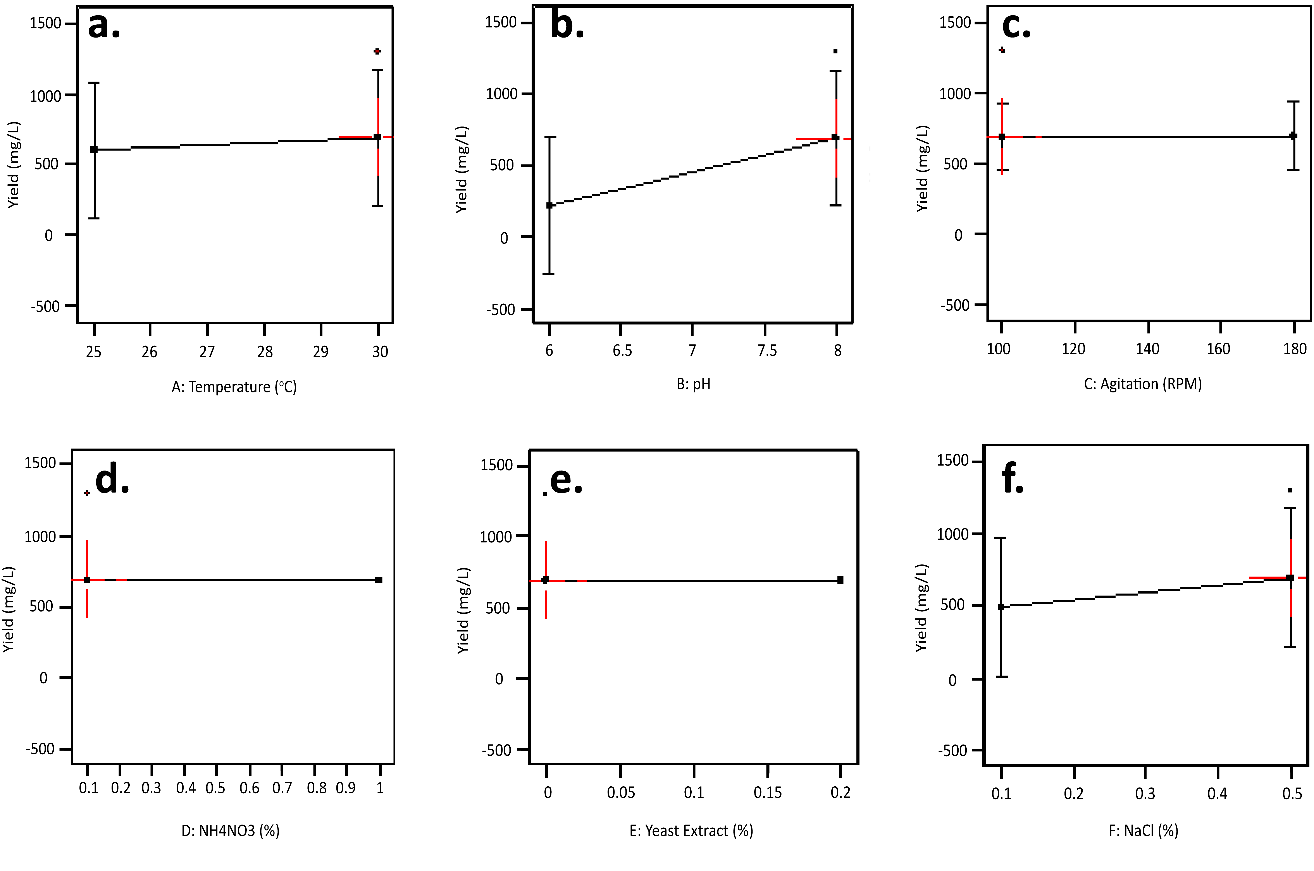
**

**Figure S5.** Significant effects of individual factors i.e. temperature, pH, agitation, NH_4_NO_3_, yeast extract and NaCl conc. (a- f) respectively, on biosurfactant production yield according to model terms.

**
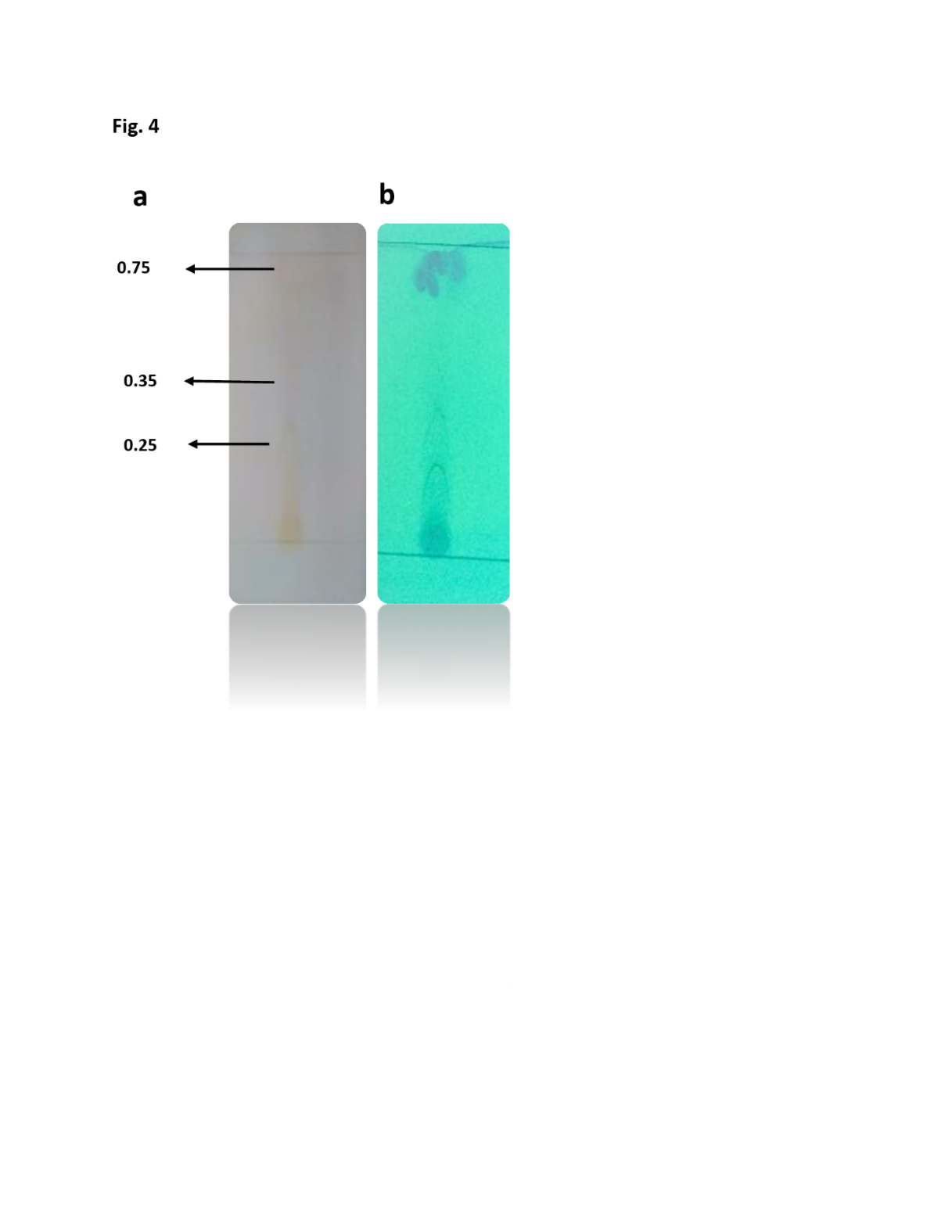
**

**Figure S6.** Thin-layer chromatography analysis of lipopeptide biosurfactant. Various reagents were applied to detect the color development figure (a) and the spots were also detect in UV figure (b).

**Table S1**. Physico-chemical and environmental parameters of collected samples.

| **S.NO** | | **Sample ID** | **Sample origin** | **Temperature (°C)** | **pH** | **Salinity (ppt)** | **TDS (mg/L)** | **Dissolved Oxygen (%)** |
| --- | --- | --- | --- | --- | --- | --- | --- | --- |
| 1 | | S1W | Surface water | 6.4 | 8.17 | 0.03 | 40.3 | 72.5 |
| 2 | | S2M | Sediment | 4 | 7.9 | 0.03 | 41.5 | 110.3 |
| 3 | | S4W | Deep water | 4.6 | 8.1 | 0.03 | 43.55 | 196.4 |
| Note: S1W= Sample 1 surface water; S2M= Sample 2 sediment/ mudd,; and S4WD= Sample 4 deep water | | | | | | | | |

**Table S2**. Screening of all 32 cultivable bacterial isolates for biosurfactants production.

| **S. No** | **Isolates** | **origin** | **Oil displacement Activity (ODA) zone diameter (cm)** | **Drop collapse technique (DCT)** | **Emulsification index (EI _24%_)** | **Surface tension measurement (SFT mN/m)** |
| --- | --- | --- | --- | --- | --- | --- |
| 1 | SIWA | Surface water | 0.0± 0 | - | 0± 0.0 | 77.7± 0.6 |
| 2 | SIWB | Surface water | NA | NA | NA | NA |
| 3 | SIWC | Surface water | 0.0± 0 | - | 30.85± 11.7 | 58.8± 0.7 |
| 4 | S1WD | Surface water | 1.2±0.3 | - | 18.75± 10.3 | 63.0± 2.5 |
| 5 | S2WE | Surface water | NA | NA | NA | NA |
| 6 | S2WF | Surface water | 0.0± 0 | + | 47.25± 10.7 | 65.6± 7.9 |
| 7 | S2WG | Surface water | NA | NA | NA | NA |
| 8 | S3WH | Surface water | 0.6± 0.3 |  | 25.95± 8.3 | 59.1± 1.2 |
| 9 | S3WI | Surface water | 1.2± 03 | + | 27.05± 10.3 | 56.7± 6.4 |
| 10 | S3WJ | Surface water | 0.0± 0 | - | 10.1± 1.6 | 78.8± 0.3 |
| 11 | S3WK1 | Surface water | 0.0± 0 | - | 0± 0.0 | 59.5± 6.2 |
| 12 | S1ML | Sediment | 0.0± 0 | - | 0± 0.0 | 68.2± 4.2 |
| 13 | S1MK2 | Sediment | 0.0± 0 | - | 0± 0.0 | 61.1± 2.8 |
| 14 | SIMM | Sediment | NA | NA | NA | NA |
| 15 | SIMN | Sediment | 0.0± 0 | - | 0± 0.0 | 76.2± 4.2 |
| 16 | SIMO | Sediment | 1.0± 0.5 | - | 0± 0.0 | 56.0± 3.8 |
| 17 | SIMP | Sediment | 0.0± 0 | - | 0± 0.0 | 56.2± 0.9 |
| 18 | S2MQ | Sediment | 0.0± 0 | - | 0± 0.0 | 56.6± 1.9 |
| 19 | S2MR | Sediment | 0.6± 0.3 | - | 0± 0.0 | 56.1± 4.3 |
| 20 | S2MS | Sediment | NA | NA | NA | NA |
| 21 | S2MT | Sediment | 4.2± 0.4 | +++ | 53.95± 0.1 | 34.15± 0.5 |
| 22 | S2MU | Sediment | 0.0± 0 | - | 0± 0.0 | 55.8± 3.5 |
| 23 | S3MV | Sediment | 0.0± 0 | - | 0± 0.0 | 75.0± 5.2 |
| 24 | S3MW | Sediment | 0.0± 0 | - | 0± 0.0 | 79.2± 1.3 |
| 25 | S3MX | Sediment | 0.0± 0 | - | 0± 0.0 | 73.0± 1.3 |
| 26 | S3MY | Sediment | 0.0± 0 | - | 0± 0.0 | 63.4± 7.4 |
| 27 | S3MZ | Sediment | 0.0± 0 | - | 0± 0.0 | 64.5± 7.4 |
| 28 | S4W1 | Deep water | 0.0± 0 | - | 0± 0.0 | 60.9± 8.8 |
| 29 | S4W2 | Deep water | 0.0± 0 | - | 0± 0.0 | 67.8± 9.1 |
| 30 | S4W3 | Deep water | 0.0± 0 | - | 0± 0.0 | 63.0± 7.7 |
| 31 | S4W4 | Deep water | 0.0± 0 | - | 0± 0.0 | 68.8± 6.6 |
| 32 | S4W5 | Deep water | 0.0± 0 | - | 30.5± 14.7 | 60.8± 9.1 |
| 33 | MSM only | --- | 0.0± 0 | - | 0± 0.0 | 78.1± 0.4 |

Note: NA= data not available; - = negative result; + = positive result; ++ = good result; +++ = best result.

**Table S3.** ANOVA table of the selected regular two-level factorial model designed for SFT (mN/m) reduction and product yield under laboratory settings.

|  |  |  | **SFT (mN/m)** |  |  |  |  |  | **Product Yield** |  |  |  |
| --- | --- | --- | --- | --- | --- | --- | --- | --- | --- | --- | --- | --- |
| **Source** | **Sum of Squares** | **df** | **Mean Square** | **F-value** | **p-value** |  | **Sum of Squares** | **df** | **Mean Square** | **F-value** | **p-value** |  |
| **Model** | 908.12 | 11 | 82.56 | 7.83 | 0.0036 | * | 2.141E+06 | 6 | 3.568E+05 | 5.80 | 0.0039 | * |
| A-Temperature | 42.58 | 1 | 42.58 | 4.04 | 0.0793 | NS | 2585.72 | 1 | 2585.72 | 0.0420 | 0.8408 | NS |
| B-pH | 189.75 | 1 | 189.75 | 18.00 | 0.0028 | * | 7.282E+05 | 1 | 7.282E+05 | 11.83 | 0.0044 | * |
| C-Agitation | 40.64 | 1 | 40.64 | 3.86 | 0.0852 | NS | 44.22 | 1 | 44.22 | 0.0007 | 0.9790 | NS |
| D-NH4NO3 | 15.02 | 1 | 15.02 | 1.42 | 0.2668 | NS | 1.284E+06 | 1 | 1.284E+06 | 20.87 | 0.0005 | ** |
| E-Yeast Extract | 164.48 | 1 | 164.48 | 15.61 | 0.0042 | * | 434.72 | 1 | 434.72 | 0.0071 | 0.9343 | NS |
| F-NaCl | 70.98 | 1 | 70.98 | 6.73 | 0.0319 | * | 1.249E+05 | 1 | 1.249E+05 | 2.03 | 0.1779 | NS |
| **Residual** | 84.32 | 8 | 10.54 | ----- | ------- | --- | 8.000E+05 | 13 | 61537.67 | ------ | ------- | ---- |
| Lack of Fit | 83.95 | 5 | 16.79 | 137.07 | 0.0010 | ** | 7.979E+05 | 10 | 79791.46 | 115.36 | 0.0012 | ** |
| Pure Error | 0.3675 | 3 | 0.1225 | ------- | ------ | --- | 2075.00 | 3 | 691.67 | ----- | ----- |  |
| **Cor Total** | 992.44 | 19 | ------ | ------- | ------- | --- | 2.941E+06 | 19 | ------ | ------- | ------- | ---- |

**Notes:** df = degree of freedom; Significance level: ** = highly significant (p < 0.001), * = significant (p < 0.05), NS = non-significant (p > 0.05)

**Table S4.** Effect of environmental factors (temperature, pH and NaCl concentrations) on (40mg/L) crude biosurfactant production by determining surface tension measurement.

| **Temperature (⁰C)** | **Surface tension (mN/m)** | **pH** | **Surface tension (mN/m)** | **NaCl conc. (%) (w/v)** | **Surface tension (mN/m)** |
| --- | --- | --- | --- | --- | --- |
| 4 | 44.2±2.5 | 3 | 40.85±0.4 | 1 | 39.15±2.9 |
| 30 | 36.5±0.6 | 6 | 36.7±0.4 | 3 | 37.6±0.6 |
| 50 | 31.9±0.3 | 7 | 38.4±3.5 | 5 | 38.25±0.5 |
| 100 | 31.5±0.1 | 8 | 39.35±1.6 | 7 | 38.4±0.4 |
| 121 | 31.55±0.4 | 10 | 39.65±1.1 | 9 | 39.65±0.6 |

**Table S5.** Molecular mass study of lipopeptide biosurfactants of *Bacillus nealsonii* (S2MT) by LC-ESI/MS.

| **S.NO** | **Obtained mass (m/z)** | **Retention time (min)** | **Family** | **Assignment** |
| --- | --- | --- | --- | --- |
| 1 | 1008.76 | 12.48 | Surfactin like | C13 [M⁺H]⁺ |
| 2 | 1030.74 | 12.48 | - | C13 [M⁺Na]⁺ |
| 3 | 1022.78 | 14.26 | - | C14 [M⁺H]⁺ |
| 4 | 1044.76 | 14.26 | - | C14 [M⁺Na]⁺ |
| 5 | 1036.78 | 15.70 | - | C15 [M⁺H]⁺ |
| 6 | 1058.77 | 15.70 | - | C15 [M⁺Na]⁺ |
